# Supplementary material for: Enhanced Lung Cancer Survival Prediction Using Semi-Supervised Pseudo-Labeling and Learning from Diverse PET/CT Datasets
Source: Cancers (Basel). 2025 Jan 17;17(2):285. doi: 10.3390/cancers17020285 (PMC11763441; doi:10.3390/cancers17020285)
Supplement: Supplementary file 1 [file cancers-17-00285-s001.zip › cancers-3295747-supplementary.pdf]

Supplemental File

1. Methods and materials

Supplemental Tabel S1. Demographic and Clinicopathologic Features of Patients

**Characteristics**	
**Source (TCIA / BC Cancer)**	32 (16%) / 167 (84%)
**Sex (Male / Female)**	99 (49%) / 100 (51%)
**Age (Mean±SD)**	Range: 24-87 (68 ±10.11)
**Surgery Category**	
**Lobectomy**	87 (43%)
**Segmentectomy**	13 (7%)
**Pneumonectomy**	4 (2%)
**Ethnicity**	
**Asian**	26 (13%)
**Neither Unknown**	173 (87%)
**Radiation Therapy**	
**Yes**	74 (37%)
**No**	31 (16%)
**Histology**	
**Non-small cell carcinoma**	24 (12%)
**Squamous cell carcinoma**	46 (23%)
**Adenocarcinoma**	109 (55%)
**Acinar cell carcinoma**	3 (1.5%)
**Neuroendocrine**	3 (1.5%)
**Combined small cell carcinoma**	1 (<1%)
**Large cell carcinoma**	1 (<1%)
**Adenosquamous**	3 (1.5%)
**Stage at diagnose**	
**IA**	32 (16%)
**IIA**	54 (27%)
**IB**	56 (28%)
**IIB**	32 (16%)
**IIIA**	3 (1.5%)
**Smoking**	166 (average of 36.4 smoking pack year)
**Chemotherapy**	
**Yes**	59 (30%)
**No**	140 (70%)
**Metastasis**	
**Yes**	64 (32%)
**No**	102 (51%)

Supplemental Tabel S2. Parameters set in different binary classification a hazard ratio survival algorithm.

Classification Algorithms	Multi-Layer Perceptron	MLPClassifier(hidden_layer_sizes=(15,(10//2),2), activation='relu', solver='adam', max_iter=100, random_state=42)
	Support Vector Machine	SVC(kernel='rbf', C=0.001, gamma='auto', random_state=42)
	K-Nearest Neighbor	KNeighborsClassifier(n_neighbors=25)
Hazard Ration Survival Analysis Algorithms	Fast Survival SVM	RandomSurvivalForest(n_estimators=10,min_samples_split=5,min_samples_leaf=10,max_features="sqrt",n_jobs=-1,random_state=42)
	Component-wise Gradient Boosting Survival Analysis	ComponentwiseGradientBoostingSurvivalAnalysis(random_state=42)

	Random Survival Forest	FastSurvivalSVM(max_iter=512, tol=1e-6, random_state=42)

1.1. Handcrafted Radiomics Features (HRF)

Radiomics features are quantitative descriptors extracted from medical images that capture tissue and tumor characteristics not visible to the naked eye. These features fall into categories such as shape, intensity, texture, and wavelet-transformed data. They are used to analyze heterogeneity, spatial patterns, and physiological properties of tumors. Radiomics has immense potential in personalized medicine, aiding in diagnosis, prognosis, and treatment response prediction. Advanced feature extraction techniques, including handcrafted methods, enhance the reliability and reproducibility of these biomarkers. Ensuring standardized extraction protocols is crucial for the clinical translation of radiomics in diverse applications. Radiomic feature generator within ViSERA has been extensively standardized in reference to the Image Biomarker Standardization Initiative (ISBI) [1]. There is a total of 487 standardized radiomics features in SERA, including 79 first-order features (morphology, statistical, histogram, and intensity-histogram features), 272 higher-order 2D features, and 136 3D features. We employed all 79 first-order features and 136 3D features such as: 29 Morphology features (Morph), 2 Local intensity features (LOC), 18 Statistics features (STAT), 23 Intensity histogram features (IH), 7 Intensity volume histogram features (IVH), 50 Co-occurrence matrix (3D, averaged and merged) features (CM), 32 Run length matrix (3D, averaged and merged) features (RLM), 16 Size zone matrix (3D) features (SZM), 16 Distance zone matrix (3D) features (DZM), 5 Neighbourhood grey tone difference matrix (3D) features (NGT), and 17 Neighbouring grey level dependence matrix (3D) features (NGL). A brief description for every radiomics feature is available in Supplemental Table S5.

1.2. Deep Radiomics Features (DRF)

Deep radiomics features are high-level, data-driven descriptors extracted from medical images using deep learning techniques. Unlike handcrafted features, which rely on predefined mathematical formulas, deep radiomics features are automatically learned by models, capturing complex, multi-scale patterns and hierarchical representations in the data. These features provide valuable insights into tumor characteristics, such as heterogeneity, morphology, and spatial distribution, which are crucial for tasks like diagnosis, prognosis, and treatment planning. They can outperform traditional radiomics in scenarios involving diverse or unstructured datasets, thanks to their ability to adaptively learn from data. However, challenges remain in the interpretability, reproducibility, and standardization of deep radiomics features for clinical use. Combining deep radiomics with traditional methods can further enhance their reliability and clinical impact.Every 3D Autoencoder typically consists of two networks: 1) an encoder network and 2) a decoder network. The decoding layer converts this representation back to the original images after the encoding layer converts the input images to a latent representation, or bottleneck. Therefore, in an Autoencoder, the number of neurons in the input and output layers must be equal. Additionally, the input data and the training label are identical. As shown in Supplemental file (Supplemental Figure S1), our network consists of three 3×3 convolutional layers, followed by a 2×2 max-pooling operation and a leaky rectified linear unit (LeakyReLU) for each layer. Parameters are reduced by using the pooling layers. Three 3×3 convolutional layers make up the decoder path, which is then followed by a LeakyReLU and an up-sampling procedure. For the suggested Autoencoder, we applied a widely used loss function termed binary cross-entropy. As a result, Adam, a gradient-based optimization technique, was used to train the suggested Autoencoder to minimize the loss function. Finally, 1024 DRFs were extracted from the bottleneck layer using the segmented CT and PET images.

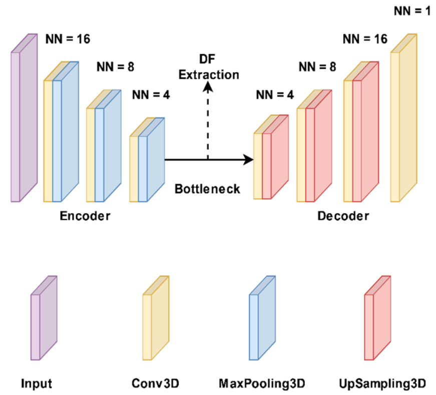

Supplemental Figure S1. Structure of our autoencoder model. It includes three 3 × 3 convolutional layers, each followed by a leaky rectified linear unit (LeakyReLU) and a 2 × 2 max-pooling operation. The decoder path includes three 3 × 3 convolutional layers, followed by a LeakyReLU and an up-sampling operation. NN: Number of neurons.

1.3. Combination of Imaging Feature datasets

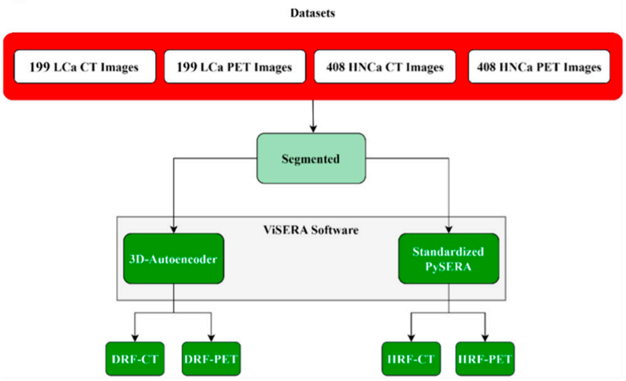

Supplemental Figure S2. Procedure of Radiomic (HRF) and deep feature (DRF) extraction from segmented PET and CT images. DRF-CT: DRFs extracted the segmented Lung (LCa) and head and neck cancer (HNCa) CT images, DRF-PET: DRFs extracted from the segmented LCa and HNCa PET images, HRF-CT: HRFs extracted from the segmented LCa and HNCa CT images, and HRF-PET: DRFs extracted the segmented LCa and HNCa PET images.

1.4. Principal Component Analysis (PCA)

PCA is a statistical unsupervised technique used in data analysis and Machine Learning (ML) to simplify the complexity of high-dimensional data while retaining most of the information. It works by identifying the directions, called principal components, along which the variability of the data is maximized. These components are orthogonal to each other and are derived from the eigenvectors of the data's covariance matrix, ranked according to their eigenvalues. In this study, PCA reduces the dimensionality by projecting the original data onto a smaller set of 10 significant components to prevent overfitting in ML prediction algorithm [2].

1.5. Classification Algorithms (CA)

ML algorithms improve task performance without requiring explicit programming. ML-based approaches seek to automatically build classification or prediction models by capturing statistically robust patterns in the analyzed data. A total of 4 known classification algorithms were used, including, Multi-Layer Perceptron (MLP) [3], Support Vector Machine (SVM) [4] K-Nearest Neighbor (KNN) [5], Ensemble Voting Algorithms (EV) were experimentally selected among various families of ML algorithms. In addition, we tuned the hyperparameters of the classifiers using 5-fold cross-validation and grid-search optimization, as elaborated in Supplemental Table S2. The grid-search optimization method has the potential to significantly increase the performance of ML algorithms. Datapoints were divided into sections five-fold cross-validation process (80% of total samples) and external nested test (remaining 20%). 5-fold cross-validation was performed to select the best HMLSs. In each fold, 4 divisions were utilized for the training and validation process.

1.6. Hazard Ratio Survival Analysis (SRA)

These kinds of algorithms are statistical methods used to analyze the expected duration of time until one or more events occur, such as death in biological organisms and failure in mechanical systems. In this study, 4 known survival prediction algorithms such as Fast Survival SVM (FSVM) [6], Component-wise Gradient Boosting Survival Analysis (CWGB) [7], Random Survival Forest (RSF) [8], and Cox Regression (COXR) [9] Algorithms were employed to survival analysis. The survival analysis used the median technique to categorize samples into low- and high-risk levels, then trained SRAs using these two categories along with their continuous time data. Additionally, the algorithms were evaluated using a nested five-fold cross-validation approach to ensure robustness and accuracy.

1.7. Pseudo-labeling

Pseudo-labeling [10] is a technique for semi-supervised learning, which is a type of ML that uses both labeled and unlabeled data. Pseudo-labeling works by using a model trained on labeled data to predict the labels for unlabeled data, and then using those “pseudo labels” to train the model in a supervised way on the unlabeled data. This can help improve the accuracy and generalization of the model, especially when there is not enough labeled data available. Of course, ultimately the performance of such a framework is tested on a fully labeled dataset.

1.8. High Dimensional Hotelling’s T Squared Test for p>>n

This method compares the means of two normal populations when the feature number (p) of component is larger than the sample number (n). Employing assumptions of the classical multivariate analysis of variance enables us to generalize Pooled Hotelling's T^2_{pooled} p-asymptotic distribution law. Two populations have different sample size (n_a and n_b are number) and identical features size (p). $\sum_p$ is covariance matrix.

For $n_a \geq 1$, $n_b \geq 1$, and $p \geq 1$, assume that:

$\{X_{ai}\}_{i=1,\ldots,n_a} \sim N_p(\mu_{pa}, \Sigma_p)$, $\{X_{bi}\}_{i=1,\ldots,n_b}\ N_p(\mu_{pb}, \Sigma_p)$, Two finite sequence are independent.

$0 < \underline{\sigma} = \lim_{p\to\infty} \frac{tr(\Sigma_p)}{p} < +\infty$, $0 < \underline{\sigma^2}\ \ \underline{\sigma} = \lim_{p\to\infty} \frac{tr(\Sigma_p{}^2)}{p} < +\infty$ and $0 < \underline{\sigma^4}\ \ \underline{\sigma} = \lim_{p\to\infty} \frac{tr(\Sigma_p{}^4)}{p} < +\infty$

To estimate the difference of the two means in two different populations, we particularly considered a confidence region. Moreover, we also test the difference of the two means by considering rejection region. We can thus define a p-asymptotic Confidence Region for difference of the means $\mu_{pa} - \mu_{pb}$:

Assumption: $n_a + n_b \geq 3$, $n = n_a + n_b - 2$, $\Delta_{mp} = \mu_{pa} - \mu_{pb}$, $p \to \infty$:

$$CR_{1-P\_value}(\mu_{pa} - \mu_{pb}) = \left\{\Delta_{mp} \in R^p : \frac{\sigma^2}{\underline{\sigma^2}} \times \frac{p}{n} \times \left(\frac{1}{n_a} + \frac{1}{n_b}\right)^{-1} \times ((\underline{X}_a - \underline{X}_b) - (\mu_{pa} - \mu_{pb}))' \times S^{+}_{pooled} \times (\underline{X}_a - \underline{X}_b) - (\mu_{pa} - \mu_{pb}))\ D \to \chi^2\ (n)\right\} \quad \text{(S1)}$$

where $\underline{X}_a$ and $\underline{X}_b$ are the two sample means, and S_{pooled} is the pooled sample covariance matrix. $S_{pooled}{}^{+}$ and χ^2 are called Moore-Penrose generalized inverse and Chi-square Distribution Table respectively.

$$S_{pooled}{}^{+} = \sum_{i:\lambda_i \neq 0} \frac{1}{\lambda_i} e_i e_i' \quad \text{(S2)}$$

where $\{\lambda_i\}_{i=1,\ldots,p}$ and $\{e_i\}_{i=1,\ldots,p}$ are the eigenvalues and eigenvectors of S_{pooled} respectively.

The p-asymptotic confidence level is defined as 1 – P_value. Moreover, we consider the following rejection region: Reject H0 in favor of H1 for a p-asymptotic Hypothesis Test of $H_0: \mu_{pa} - \mu_{pb} = \Delta\mu_{0p}$ versus $H1: \mu_{pa} - \mu_{pb} \neq \Delta\mu_{0p}$ with p-asymptotic significance level P_Value [11].

2. Results

Supplemental Tabel S3. The external nested testing performances providing from Hybrid Machine Learning Systems (Principal Component Analysis linked with classifiers) while applied on DRF-CT: DRFs (deep Radiomic features) extracted the segmented CT images, DRF-PET: DRFs extracted the segmented PET images, HRF-CT: HRFs (handcrafted Radiomic features) extracted the segmented CT images and HRF-PET: HRFs extracted the segmented PET images. MLP: Multi-Layer Perceptron; SVM: Support Vector Machine, BR: Bagging Regression, KNN: K-Nearest Neighbor, EV: Ensemble Voting. FV_AAC: Average Accuracy in External Nested Test, STD: Standard Deviation.

Feature	Modalities	Type	MLP		SVM		KNN		EV		XGB		LGB	
			FV_Mean Acc (STD)	EX_Mean Acc (STD)	FV_Mean Acc (STD)	EX_Mean Acc (STD)	FV_Mean Acc (STD)	EX_Mean Acc (STD)	FV_Mean Acc (STD)	EX_Mean Acc (STD)	FV_Mean Acc (STD)	EX_Mean Acc (STD)	FV_Mean Acc (STD)	EX_Mean Acc (STD)
HRF	CT	SSL	0.75 (0.06)	0.68 (0.03)	0.73 (0.07)	0.68 (0.0)	0.72 (0.06)	0.68 (0.01)	0.72 (0.06)	0.68 (0.02)	0.76 (0.06)	0.62 (0.01)	0.75 (0.05)	0.6 (0.0)
HRF	CT	SL	0.27 (0.07)	0.32 (0.0)	0.57 (0.09)	0.59 (0.06)	0.56 (0.06)	0.58 (0.03)	0.57 (0.05)	0.55 (0.01)	0.70 (0.06)	0.63 (0.02)	0.71 (0.04)	0.64 (0.05)
HRF	PET	SSL	0.77 (0.10)	0.72 (0.02)	0.76 (0.10)	0.72 (0.01)	0.76 (0.1)	0.72 (0.01)	0.73 (0.07)	0.68 (0.02)	0.74 (0.05)	0.67 (0.05)	0.72 (0.05)	0.7 (0.00)
HRF	PET	SL	0.28 (0.10)	0.28 (0.0)	0.6 (0.07)	0.59 (0.03)	0.62 (0.04)	0.61 (0.05)	0.58 (0.05)	0.60 (0.01)	0.69 (0.07)	0.62 (0.02)	0.68 (0.04)	0.64 (0.06)
DRF	CT	SSL	0.83 (0.06)	0.79 (0.01)	0.82 (0.06)	0.77 (0.02)	0.81 (0.06)	0.74 (0.01)	0.69 (0.04)	0.72 (0.01)	0.7 (0.06)	0.66 (0.04)	0.71 (0.07)	0.75 (0.00)
DRF	CT	SL	0.44 (0.06)	0.39 (0.01)	0.62 (0.02)	0.60 (0.09)	0.65 (0.08)	0.64 (0.06)	0.64 (0.03)	0.65 (0.01)	0.67 (0.01)	0.58 (0.06)	0.69 (0.06)	0.57 (0.04)
DRF	PET	SSL	0.85 (0.05)	0.80 (0.01)	0.81 (0.06)	0.80 (0.01)	0.83 (0.06)	0.81 (0.04)	0.79 (0.06)	0.82 (0.01)	0.66 (0.08)	0.74 (0.05)	0.71 (0.08)	0.74 (0.02)
DRF	PET	SL	0.50	0.50	0.59	0.55	0.60	0.52	0.60	0.62	0.61	0.68	0.62	0.68

			(0.05)	(0.01)	(0.10)	(0.05)	(0.05)	(0.04)	(0.04)	(0.01)	(0.06)	(0.04)	(0.09)	(0.03)
DRF + HRF	CT	SSL	0.74 (0.05)	0.70 (0.02)	0.74 (0.05)	0.70 (0.03)	0.73 (0.05)	0.71 (0.02)	0.72 (0.05)	0.72 (0.02)	0.64 (0.05)	0.72 (0.05)	0.5 (0.23)	0.55 (0.22)
DRF + HRF	CT	SL	0.31 (0.05)	0.30 (0.01)	0.59 (0.06)	0.57 (0.03)	0.63 (0.09)	0.57 (0.05)	0.59 (0.04)	0.58 (0.02)	0.6 (0.03)	0.74 (0.04)	0.62 (0.0)	0.74 (0.06)
DRF + HRF	PET	SSL	0.78 (0.09)	0.75 (0.01)	0.77 (0.08)	0.75 (0.02)	0.77 (0.07)	0.76 (0.01)	0.76 (0.05)	0.75 (0.02)	0.68 (0.08)	0.78 (0.03)	0.71 (0.08)	0.75 (0.0)
DRF + HRF	PET	SL	0.49 (0.06)	0.41 (0.02)	0.55 (0.07)	0.53 (0.08)	0.60 (0.07)	0.59 (0.06)	0.60 (0.04)	0.62 (0.02)	0.61 (0.05)	0.68 (0.06)	0.62 (0.06)	0.69 (0.07)

Supplemental Tabel S4. The external nested testing performances providing from Hybrid Machine Learning Systems (Principal Component Analysis linked with hazard ratio survival analysis algorithms (SRA)) while applied on DRF-CT: DRFs (deep Radiomic features) extracted the segmented CT images, DRF-PET: DRFs extracted the segmented PET images, HRF-CT: HRFs (handcrafted Radiomic features) extracted the segmented CT images and HRF-PET: HRFs extracted the segmented PET images. FSVM: Fast Survival Support Vector Machine, CWGB: Component-wise Gradient Boosting Survival Analysis, RSF: Random Survival Forest, and COXR: Cox Regression. FV_AAC: Average Accuracy in External Nested Test, STD: Standard Deviation.

Dataset	SRA	FV_AAC (STD)	EX_AAC (STD)	P-value
HRF-CT	RSF	0.65 (0.04)	0.66 (0.05)	0.0025
HRF-PET	RSF	0.62 (0.10)	0.61 (0.04)	0.0011
HRF-CT	FSVM	0.54 (0.06)	0.62 (0.23)	0.14
HRF-PET	FSVM	0.54 (0.10)	0.64 (0.07)	0.59
HRF-CT	CWGB	0.79 (0.08)	0.80 (0.00)	0.0000025
HRF-PET	CWGB	0.62 (0.05)	0.59 (0.03)	0.05
HRF-CT	COXR	0.57 (0.09)	0.66 (0.17)	0.002
HRF-PET	COXR	0.57 (0.11)	0.67 (0.09)	0.23
DRF-CT	RSF	0.59 (0.10)	0.66 (0.06)	0.03
DRF-PET	RSF	0.58 (0.06)	0.58 (0.08)	0.04
DRF-CT	FSVM	0.59 (0.05)	0.66 (0.05)	0.04
DRF-PET	FSVM	0.56 (0.10)	0.62 (0.04)	0.01
DRF-CT	CWGB	0.80 (0.10)	0.80 (0.00)	0.0000039
DRF-PET	CWGB	0.53 (0.09)	0.59 (0.03)	0.12
DRF-CT	COXR	0.55 (0.06)	0.66 (0.07)	0.008
DRF-PET	COXR	0.51 (0.07)	0.62 (0.05)	0.003
DRF-CT plus HRF-CT	RSF	0.6 (0.04)	0.79 (0.05)	0.003
DRF-PET plus HRF-PET	RSF	0.56 (0.10)	0.71 (0.04)	0.001
DRF-CT plus HRF-CT	FSVM	0.61 (0.08)	0.8 (0.00)	0.000003
DRF-PET plus HRF-PET	FSVM	0.63 (0.05)	0.76 (0.03)	0.05
DRF-CT plus HRF-CT	CWGB	0.58 (0.06)	0.62 (0.23)	0.14
DRF-PET plus HRF-PET	CWGB	0.58 (0.10)	0.64 (0.07)	0.59
DRF-CT plus HRF-CT	COXR	0.57 (0.09)	0.66 (0.17)	0.002
DRF-PET plus HRF-PET	COXR	0.57 (0.11)	0.67 (0.09)	0.23

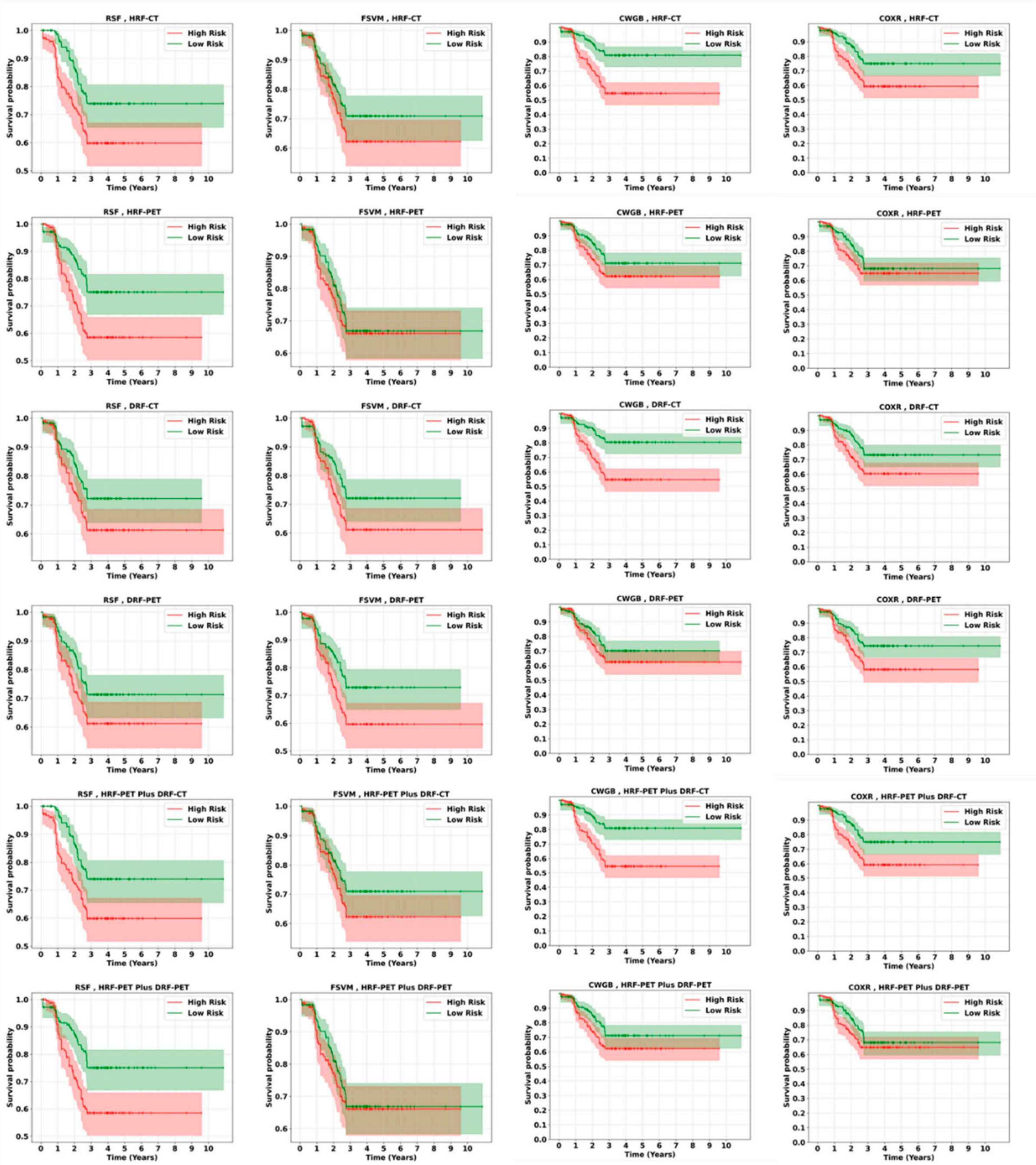

Supplemental Figure S3. Variety of Kaplan-Meier survival curves generated by Principal Component Analysis linked with three hazard ratio survival analysis algorithms, applied on DRF-CT: DRFs (deep Radiomic features) extracted the segmented CT images, DRF-PET: DRFs extracted the segmented PET images, HRF-CT: HRFs (handcrafted Radiomic features) extracted the segmented CT images and HRF-PET: HRFs extracted the segmented PET images. FSVM: Fast Survival Support Vector Machine, CWGB: Component-wise Gradient Boosting Survival Analysis, RSF: Random Survival Forest, and COXR: Cox Regression.

Supplemental Table S5. Radiomics features used in the study

Family	Image Biomarker	Family	Image Biomarker
**Morphology**	Volume (mesh-based)	Co-occurrence matrix (3D, merged)	Joint entropy

**Morphology**	Volume (counting)	Co-occurrence matrix (3D, merged)	Difference average
**Morphology**	Surface area	Co-occurrence matrix (3D, merged)	Difference variance
**Morphology**	Surface to volume ratio	Co-occurrence matrix (3D, merged)	Difference entropy
**Morphology**	Compactness 1	Co-occurrence matrix (3D, merged)	Sum average
**Morphology**	Compactness 2	Co-occurrence matrix (3D, merged)	Sum variance
**Morphology**	Spherical disproportion	Co-occurrence matrix (3D, merged)	Sum entropy
**Morphology**	Sphericity	Co-occurrence matrix (3D, merged)	Angular second moment
**Morphology**	Asphericity	Co-occurrence matrix (3D, merged)	Contrast
**Morphology**	Centre of mass shift	Co-occurrence matrix (3D, merged)	Dissimilarity
**Morphology**	Maximum 3D diameter	Co-occurrence matrix (3D, merged)	Inverse difference
**Morphology**	Major axis length	Co-occurrence matrix (3D, merged)	Inverse difference normalised
**Morphology**	Minor axis length	Co-occurrence matrix (3D, merged)	Inverse difference moment
**Morphology**	Least axis length	Co-occurrence matrix (3D, merged)	Inverse difference moment normalised
**Morphology**	Elongation	Co-occurrence matrix (3D, merged)	Inverse variance
**Morphology**	Flatness	Co-occurrence matrix (3D, merged)	Correlation
**Morphology**	Volume density (AABB)	Co-occurrence matrix (3D, merged)	Autocorrelation
**Morphology**	Area density (AABB)	Co-occurrence matrix (3D, merged)	Cluster tendency
**Morphology**	Volume density (OMBB)	Co-occurrence matrix (3D, merged)	Cluster shade
**Morphology**	Area density (OMBB)	Co-occurrence matrix (3D, merged)	Cluster prominence
**Morphology**	Volume density (AEE)	Co-occurrence matrix (3D, merged)	Information correlation 1
**Morphology**	Area density (AEE)	Co-occurrence matrix (3D, merged)	Information correlation 2
**Morphology**	Volume density (MVEE)	Run length matrix (3D, averaged)	Short runs emphasis
**Morphology**	Area density (MVEE)	Run length matrix (3D, averaged)	Long runs emphasis
**Morphology**	Volume density (convex hull)	Run length matrix (3D, averaged)	Low grey level run emphasis
**Morphology**	Area density (convex hull)	Run length matrix (3D, averaged)	High grey level run emphasis
**Morphology**	Integrated intensity	Run length matrix (3D, averaged)	Short run low grey level emphasis
**Morphology**	Moran's I index	Run length matrix (3D, averaged)	Short run high grey level emphasis
**Morphology**	Geary's C measure	Run length matrix (3D, averaged)	Long run low grey level emphasis
**Local intensity**	Local intensity peak	Run length matrix (3D, averaged)	Long run high grey level emphasis
**Local intensity**	Global intensity peak	Run length matrix (3D, averaged)	Grey level non-uniformity
**Statistics**	Mean	Run length matrix (3D, averaged)	Grey level non-uniformity normalised
**Statistics**	Variance	Run length matrix (3D, averaged)	Run length non-uniformity
**Statistics**	Skewness	Run length matrix (3D, averaged)	Run length non-uniformity normalised
**Statistics**	(Excess) kurtosis	Run length matrix (3D, averaged)	Run percentage
**Statistics**	Median	Run length matrix (3D, averaged)	Grey level variance

**Statistics**	Minimum	Run length matrix (3D, averaged)	Run length variance
**Statistics**	10th percentile	Run length matrix (3D, averaged)	Run entropy
**Statistics**	90th percentile	Run length matrix (3D, merged)	Short runs emphasis
**Statistics**	Maximum	Run length matrix (3D, merged)	Long runs emphasis
**Statistics**	Interquartile range	Run length matrix (3D, merged)	Low grey level run emphasis
**Statistics**	Range	Run length matrix (3D, merged)	High grey level run emphasis
**Statistics**	Mean absolute deviation	Run length matrix (3D, merged)	Short run low grey level emphasis
**Statistics**	Robust mean absolute deviation	Run length matrix (3D, merged)	Short run high grey level emphasis
**Statistics**	Median absolute deviation	Run length matrix (3D, merged)	Long run low grey level emphasis
**Statistics**	Coefficient of variation	Run length matrix (3D, merged)	Long run high grey level emphasis
**Statistics**	Quartile coefficient of dispersion	Run length matrix (3D, merged)	Grey level non-uniformity
**Statistics**	Energy	Run length matrix (3D, merged)	Grey level non-uniformity normalised
**Statistics**	Root mean square	Run length matrix (3D, merged)	Run length non-uniformity
**Intensity histogram**	Mean	Run length matrix (3D, merged)	Run length non-uniformity normalised
**Intensity histogram**	Variance	Run length matrix (3D, merged)	Run percentage
**Intensity histogram**	Skewness	Run length matrix (3D, merged)	Grey level variance
**Intensity histogram**	Kurtosis	Run length matrix (3D, merged)	Run length variance
**Intensity histogram**	Median	Run length matrix (3D, merged)	Run entropy
**Intensity histogram**	Minimum	Size zone matrix (3D)	Small zone emphasis
**Intensity histogram**	10th percentile	Size zone matrix (3D)	Large zone emphasis
**Intensity histogram**	90th percentile	Size zone matrix (3D)	Low grey level emphasis
**Intensity histogram**	Maximum	Size zone matrix (3D)	High grey level emphasis
**Intensity histogram**	Mode	Size zone matrix (3D)	Small zone low grey level emphasis
**Intensity histogram**	Interquartile range	Size zone matrix (3D)	Small zone high grey level emphasis
**Intensity histogram**	Range	Size zone matrix (3D)	Large zone low grey level emphasis
**Intensity histogram**	Mean absolute deviation	Size zone matrix (3D)	Large zone high grey level emphasis
**Intensity histogram**	Robust mean absolute deviation	Size zone matrix (3D)	Grey level non-uniformity
**Intensity histogram**	Median absolute deviation	Size zone matrix (3D)	Grey level non uniformity normalised
**Intensity histogram**	Coefficient of variation	Size zone matrix (3D)	Zone size non-uniformity
**Intensity histogram**	Quartile coefficient of dispersion	Size zone matrix (3D)	Zone size non-uniformity normalised
**Intensity histogram**	Entropy	Size zone matrix (3D)	Zone percentage
**Intensity histogram**	Uniformity	Size zone matrix (3D)	Grey level variance
**Intensity histogram**	Maximum histogram gradient	Size zone matrix (3D)	Zone size variance
**Intensity histogram**	Maximum gradient grey level	Size zone matrix (3D)	Zone size entropy
**Intensity histogram**	Minimum histogram gradient	Distance zone matrix (3D)	Small distance emphasis

**Intensity histogram**	Minimum gradient grey level	Distance zone matrix (3D)	Large distance emphasis
**Intensity volume histogram**	Volume fraction at 10% intensity	Distance zone matrix (3D)	Low grey level emphasis
**Intensity volume histogram**	Volume fraction at 90% intensity	Distance zone matrix (3D)	High grey level emphasis
**Intensity volume histogram**	Intensity at 10% volume	Distance zone matrix (3D)	Small distance low grey level emphasis
**Intensity volume histogram**	Intensity at 90% volume	Distance zone matrix (3D)	Small distance high grey level emphasis
**Intensity volume histogram**	Volume fraction difference between 10% and 90% intensity	Distance zone matrix (3D)	Large distance low grey level emphasis
**Intensity volume histogram**	Intensity difference between 10% and 90% volume	Distance zone matrix (3D)	Large distance high grey level emphasis
**Intensity volume histogram**	Area under the IVH curve	Distance zone matrix (3D)	Grey level non-uniformity
**Co-occurrence matrix (3D, averaged)**	Joint maximum	Distance zone matrix (3D)	Grey level non-uniformity normalised
**Co-occurrence matrix (3D, averaged)**	Joint average	Distance zone matrix (3D)	Zone distance non-uniformity
**Co-occurrence matrix (3D, averaged)**	Joint variance	Distance zone matrix (3D)	Zone distance non-uniformity normalised
**Co-occurrence matrix (3D, averaged)**	Joint entropy	Distance zone matrix (3D)	Zone percentage
**Co-occurrence matrix (3D, averaged)**	Difference average	Distance zone matrix (3D)	Grey level variance
**Co-occurrence matrix (3D, averaged)**	Difference variance	Distance zone matrix (3D)	Zone distance variance
**Co-occurrence matrix (3D, averaged)**	Difference entropy	Distance zone matrix (3D)	Zone distance entropy
**Co-occurrence matrix (3D, averaged)**	Sum average	Neighbourhood grey tone difference matrix (3D)	Coarseness
**Co-occurrence matrix (3D, averaged)**	Sum variance	Neighbourhood grey tone difference matrix (3D)	Contrast
**Co-occurrence matrix (3D, averaged)**	Sum entropy	Neighbourhood grey tone difference matrix (3D)	Busyness
**Co-occurrence matrix (3D, averaged)**	Angular second moment	Neighbourhood grey tone difference matrix (3D)	Complexity
**Co-occurrence matrix (3D, averaged)**	Contrast	Neighbourhood grey tone difference matrix (3D)	Strength
**Co-occurrence matrix (3D, averaged)**	Dissimilarity	Neighbouring grey level dependence matrix (3D)	Low dependence emphasis
**Co-occurrence matrix (3D, averaged)**	Inverse difference	Neighbouring grey level dependence matrix (3D)	High dependence emphasis
**Co-occurrence matrix (3D, averaged)**	Inverse difference normalised	Neighbouring grey level dependence matrix (3D)	Low grey level count emphasis
**Co-occurrence matrix (3D, averaged)**	Inverse difference moment	Neighbouring grey level dependence matrix (3D)	High grey level count emphasis
**Co-occurrence matrix (3D, averaged)**	Inverse difference moment normalised	Neighbouring grey level dependence matrix (3D)	Low dependence low grey level emphasis
**Co-occurrence matrix (3D, averaged)**	Inverse variance	Neighbouring grey level dependence matrix (3D)	Low dependence high grey level emphasis
**Co-occurrence matrix (3D, averaged)**	Correlation	Neighbouring grey level dependence matrix (3D)	High dependence low grey level emphasis
**Co-occurrence matrix (3D, averaged)**	Autocorrelation	Neighbouring grey level dependence matrix (3D)	High dependence high grey level emphasis
**Co-occurrence matrix (3D, averaged)**	Cluster tendency	Neighbouring grey level dependence matrix (3D)	Grey level non-uniformity
**Co-occurrence matrix (3D, averaged)**	Cluster shade	Neighbouring grey level dependence matrix (3D)	Grey level non-uniformity normalised
**Co-occurrence matrix (3D, averaged)**	Cluster prominence	Neighbouring grey level dependence matrix (3D)	Dependence count non-uniformity
**Co-occurrence matrix (3D, averaged)**	Information correlation 1	Neighbouring grey level dependence matrix (3D)	Dependence count non-uniformity normalised
**Co-occurrence matrix (3D, averaged)**	Information correlation 2	Neighbouring grey level dependence matrix (3D)	Dependence count percentage
**Co-occurrence matrix (3D, merged)**	Joint maximum	Neighbouring grey level dependence matrix (3D)	Grey level variance
**Co-occurrence matrix (3D, merged)**	Joint average	Neighbouring grey level dependence matrix (3D)	Dependence count variance
**Co-occurrence matrix (3D, merged)**	Joint variance	Neighbouring grey level dependence matrix (3D)	Dependence count entropy

Supplemental Tabel S6. Table for percentage of the variance explained by just 10 principal components.

Dataset	% of the variance explained by 10 PCA features
HRF PET	88.2
HRF CT	87.1
DRF PET	89.7
DRF CT	91.5
HRF + DRF PET	85.0
HRF + DRF CT	86.7

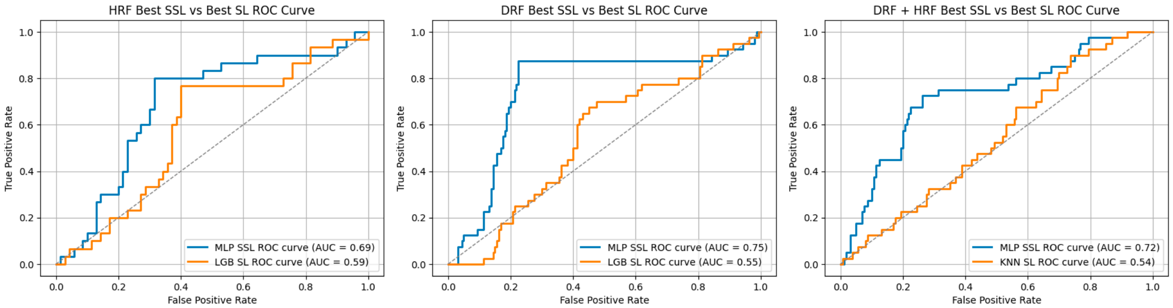

Supplemental Figure S4. ROC curves for best results applied on DRFs (deep Radiomic features), HRFs (handcrafted Radiomic features), MLP: Multi-Layer Perceptron, KNN: K-Nearest Neighbor, LGB: Light Gradient Boosting, AUC: Area Under Curve, SSL : Semi-Supervised Learning, SL: Supervised Learning

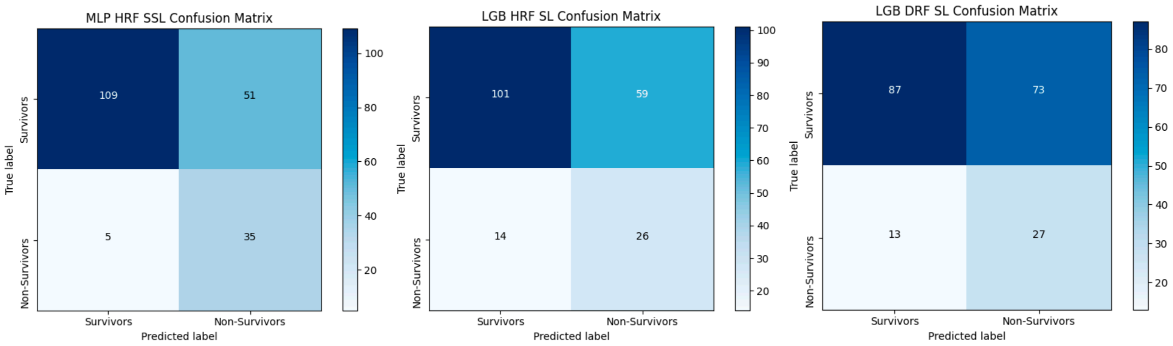

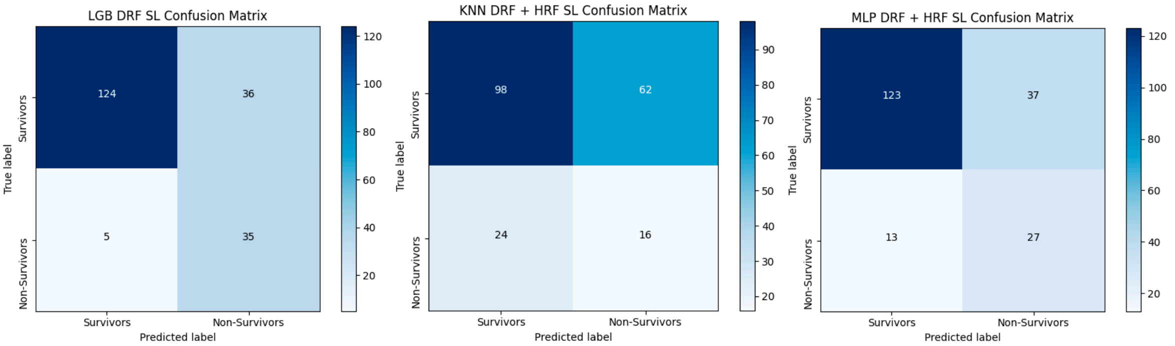

Supplemental Figure S5. Confusion Matrix for best results applied on DRFs (deep Radiomic features), HRFs (handcrafted Radiomic features), MLP: Multi-Layer Perceptron, KNN: K-Nearest Neighbor, LGB: Light Gradient Boosting, AUC: Area Under Curve, SSL : Semi-Supervised Learning, SL: Supervised Learning

References

[1] A. Zwanenburg, M. Vallières and et al, "The image biomarker standardization initiative: Standardized quantitative radiomics for high-throughput image-based phenotyping," *Radiology,* vol. 295, no. 2, pp. 328-338, 5 2020.

[2] M. Salmanpour, M. Bakhtiyari and et al, "Application of novel hybrid machine learning systems and radiomics features for non-motor outcome prediction in Parkinson's disease," *Physics in Medicine & Biology,* vol. 68, no. 3, p. 035004, 2023.

[3] G. Cybenko, "Approximation by superpositions of a sigmoidal function," *Math. Control Signal Systems,* vol. 2, no. 4, p. 303–314, 1989.

[4] C. Cortes and V. Vapnik, "Support-vector networks," *Machine Learning.,* vol. 20, no. 3, p. 273–297, 1995.

[5] L. Peterson, "K-nearest neighbor," *Scholarpedia,* vol. 4, no. 2, p. 1883, 2009.

[6] S. Pölsterl, "A Library for Time-to-Event Analysis Built on Top of scikit-learn," *Journal of Machine Learning Research,* vol. 21, no. 212, p. 1–6, 2020.

[7] S. Pölsterl, "Gradient Boosted Models," 2020. [Online]. Available: https://scikit-survival.readthedocs.io/en/stable/user_guide/boosting.html. [Accessed 9 January 2024].

[8] S. Pölsterl, "Random survival forests," 2020. [Online]. Available: https://scikit-survival.readthedocs.io/en/stable/user_guide/random-survival-forest.html. [Accessed 9 January 2024].

[9] M. Du, D. Haag and et al, "Comparison of the tree-based machine learning algorithms to Cox regression in predicting the survival of oral and pharyngeal cancers: analyses based on SEER database," *Cancers,* vol. 12, no. 10, p. 2802, 2020.

[10] P. Cascante-Bonilla, F. Tan and et al, "Curriculum Labeling: Revisiting Pseudo-Labeling for Semi-Supervised Learning," *Proceedings of the AAAI Conference on Artificial Intelligence,* vol. 35, no. 8, pp. 6912-6920, 2021.

[11] P. Secchi, A. Stamm and S. Vantini, "Inference for the mean of large p small n data: A finite-sample high-dimensional generalization of Hotelling's theorem," *Electronic Journal of Statistics,* vol. 7, pp. 2005-2031, 2013.
